# A comparative study on renewable and traditional electricity: The influence of the European Union framework and the impact of COVID-19

**DOI:** 10.1371/journal.pone.0277088

**Published:** 2022-11-21

**Authors:** Florin Teodor Boldeanu, José Antonio Clemente-Almendros, Luis Alberto Seguí-Amortegui, Constantin Duguleana

**Affiliations:** 1 Faculty of Economic Sciences and Business Administration, Department of Finance, Accounting and Economic Theory, Transilvania University of Brasov, Brașov, Romania; 2 Business and Communication Faculty, International University of La Rioja, Logroño, Spain; Zhejiang University of Finance and Economics, CHINA

## Abstract

By means of the event study approach, we analyse the effect of COVID-19 on listed European renewable and traditional electricity companies, inside and outside the European Union, for the pandemic announcement and lockdowns. We find that the pandemic negatively affected both subsectors of electricity production, but the negative effect was more intense for renewable electricity companies, since they represent a riskier investment. Moreover, this negative effect was larger for European electricity companies than for companies from countries that do not belong to the European Union. Our results show the riskier profile of the clean energy industry together with the importance of a stable and supportive regulatory framework to develop and consolidate renewable energy. Our findings have important implications for policymakers. In addition to the intrinsic risks associated with renewable energy, this type of investment poses policy and regulatory risks, which they should take into account when evaluating future energy policies. Policymakers must be aware of the importance of these specific risks, and seek to respond to investors’ expectations about long-term, stable regulations.

## Introduction

Pandemics have major repercussions for the worldwide economy [1]. COVID-19, which was declared a global pandemic by the World Health Organization (WHO) in the afternoon of 11^th^ March 2020, has had an unprecedented impact on the global economy, through the unparalleled limits on and reduction of peoples’ mobility, manufacturing activity and the consumption of electricity [2]. Investors’ confidence and financial markets suffered as a result, contributing to a observable and perceptible uncertainty about potential losses [3, 4].

The unprecedented COVID-19 outbreak created confusion and uncertainty and placed an enormous burden on public health systems and the public administration, while the level of activity in many sectors dropped. It was not only the public finances and sectorial economic activity that were affected. Pandemics tend to have an effect on investor confidence [5], which may result in financial market volatility of the companies’ return [6]. Due to the uniqueness of the COVID-19 pandemic, it provides a first-of-its-kind framework for understanding how pandemics affect the global economy and studying the impact on financial markets helps to explain that influence [7]. Moreover, as the impact is not uniform among industries, a more in-depth analysis is necessary for a more comprehensive grasp of the situation. That is why previous studies have analysed the response of financial markets to COVID-19 [3, 4, 8]. The renewable energy sector is a worldwide strategic industry [9]. The share of coal is set to decline in the future, and renewable energy will lead the transition towards a new energy model, with the greatest growth rates of demand [10]. Nevertheless, this industry is not free of risk in its return, since it suffers from high volatility [11]. The impact of COVID-19 on energy stock prices and then on companies’ return is basically due because of two factors, a reduction in the energy demand because of the mobility and production restrictions, and the uncertainty of the worldwide economy and then companies’ profitability [12]. It was found that this uncertainty negatively affects energy market prices [13]. However, even though the impact of COVID-19 on the stock prices and companies’ return in the energy sector has been analysed in the literature [14–16], as far as we know, there is no empirical evidence on the impact of COVID-19 on the electricity sector, and at the same time considering the differences between the traditional way of producing electricity and renewable electricity.

The European Union (EU) characterizes itself as a world leader and promoter of renewable energy, with its electric grid being one of the most interconnected power networks in the world. The EU institutional framework and legislation helps mitigate risk in the field of renewable electricity (e.g. volatility, break even, uncertainty of the return, high capital expenditure). In this vein, the “Europeanization” of former Eastern European countries such as the Czech Republic has helped in developing renewable electricity resources, giving rise to a five-fold increase in their capacity [17]. The continued development in the field of renewable energy resources in electricity production has a direct effect in terms of cutting CO_2_ emissions. This effect is evident in Europe, US and the wider world [18]. The EU has adopted structural and institutional changes that have had an impressive impact on the single electricity market compared to other regions, taking into account the few econometric studies that have compared EU countries with non-European countries (e.g. the USA) [19].

In the context of the European framework, the EU developed an Action Plan that aims to ensure long-term competitiveness. The transition to a low-carbon economy is a key factor for EU competitiveness. In this vein, the EU provides economic and political support for the development of the renewable energy sector. The so-called “European Green Deal” involves the target of achieving zero emissions of greenhouse gases in the EU by 2050, which represents a clear incentive to produce energy in a greener way. Nevertheless, the fact that the EU still depends on imports for energy [20] may jeopardize its strategy in this respect. In the heating sector for example, Europe is a net importer of gas from non-EU countries, which is not only an environmental issue but also an energy security issue [21]. However, while the EU promotes green energy, European countries outside the EU still base their economies on traditional ways of producing energy, using it as a geo-political tool [22].

Even though there is a large literature about the consequences of COVID-19, there is little electricity sectorial focus on financial markets and stock prices [3]. Furthermore, to the best of our knowledge, our study is the first to analyse stock price reactions and company return in the electricity sector for European companies, distinguishing on the one hand between EU and non-EU companies, and on the other between traditional and renewable electricity companies.

The aim of this paper is to analyse the impact of COVID-19 on European financial markets, further distinguishing between EU and non-EU countries. In particular, we study listed European traditional and renewable utility companies. The latter captures clean electricity, whereas the former includes electricity producers and distributors (along with nuclear and non-nuclear). Our sample consists of European electric utilities. We classified them as renewable and traditional electricity companies to analyse, from the adjusted daily market prices, the resulting cumulative average abnormal returns (CAARs) for two events related to COVID-19, the day after the WHO announcement of a global pandemic together with the start of national lockdowns in European countries. We analyse before and after the impact of both events for EU and non-EU countries using the event study method [23].

Our empirical results show that COVID-19 negatively affected listed European electricity companies, since the pandemic significantly reduced worldwide mobility and the global economy. However, this impact was heterogeneous. This negative impact was larger for renewable than for traditional electricity companies. Renewable electricity endowment displays a riskier profile due to the intrinsic characteristics of such financial endeavours. COVID-19 particularly affected the renewable sector since the disruption to the supply chain of components delayed operations in renewable projects. However, the larger negative effect on renewable electricity companies has been mitigated by the stable and supportive regulatory framework of the EU. Investments in renewables also suffer from policy risk, needing support from policymakers together with a transparent, long-term orientated regulatory framework.

Our study contributes to the literature in different ways. First, clean energy is a key sector worldwide, in Europe, particularly in the EU [24]. Even though it has an increasing proportion in the energy mix year by year, clean energy has associated intrinsic risks, among others, variability, unreliable predictability, weather dependency [25]. With the increasing importance of this type of energy is thus critical to understand the investment characteristics, risk profile and investors attitudes of renewable projects. We confirm by means of the event study methodology and using two unprecedented worldwide and national events, the WHO announcement and the pandemic lockdowns, the riskier profile of this investment. Second, due to its strategic importance for the EU, together with the importance of understanding the specific characteristics of this type of energy, it is essential to check the influence of the EU framework. In addition to confirming the different profile of both types of energy, in line with the existing empirical evidence, utilizing the event study methodology and the unique events related to COVID-19 pandemic, we show that the institutional support that renewable energy sector needs [11, 26–28] provided by the EU, reduced the financial markets uncertainty in the two events analysed. Third, even though operating conditions for companies that belong to the same sector are highly correlated with the economic environment [29] and they are assumed to be equally affected, the aim of our paper is to show that the impact may not be equal for companies working in the same sector (traditional and renewable electricity in our research).

The following sections of the article are structured as follows. In the Literature Review section, we provide a brief review of the relevant literature and develop our hypotheses. In the Methodology section, we describe the methodology used in our study. In the Data Description section, we detail the data collection process. In the Results and Discussion section, we present the results and then discuss them. Finally, in the Conclusions and policy implications section, we set out our conclusions and main policy implications.

## Literature review

There is empirical evidence about the impact of epidemics and pandemics in financial markets and in specific sectors. The 2006 SARS epidemic affected different sectors in Asian countries, such as tourism, retail, and air transportation [30–32]. Ebola significantly affected equities (e.g. mutual funds) in African countries, resulting in a massive withdrawal of savings [33]. Mctier, Tse, and Wald [34] found that flu negatively impacted stock exchange returns in the USA. Several studies analysed the effect of COVID-19 pandemic on market stock prices and returns [35]. Similar to the COVID-19 pandemic announcement, there is empirical evidence in the literature of the influence of lockdowns caused by this outbreak on stock market performance. Most of the studies showed a relationship between national lockdowns and a decline in the financial market’s liquidity, performance, and stability [36].

However, there is also empirical evidence showing that the impact is not equal for all sectors of the economy but differs depending on the industry [3]. SARS did not have a significant impact on the manufacturing industry [7]. COVID-19 negatively affected Chinese financial markets, but different sectors were found to show diverse reactions to the pandemic [4]. Related to the energy sector, Nguyen [14] found that energy sectors suffered a substantial negative abnormal return. Ramelli and Wagner [16] documented that energy and consumer services industries experienced the largest impact. Mazur, Dang and Vega [37] found important differences in the market prices reaction across sectors. However, to best of our knowledge, there is no empirical evidence about the impact of COVID-19 for the different types of energy, traditional and renewable, together with the effect of COVID-19 pandemic in these two types of energy depending on the institutional framework.

The support for greener electricity production based on renewable sources is a priority of the EU energy goals [2, 38]. The sustainable transition to a low-carbon and more resource-efficient economy are strategic objectives to ensure the long-term competitiveness of the EU economy. In this context, there is a need to adapt public policies to this new reality; more specifically, the financial system plays a key role. To ensure sustainable economic growth, the EU needs to encourage the financial system to realign private capital to more sustainable investment, fostering a stable, transparent and long-term financial framework [39]. Indeed, this is the core of the EU’s Capital Markets Union. Accordingly, the EU has developed an Action Plan on sustainable finance, adopted by the European Commission in March 2018, to link finance to the needs and challenges of the European economy. This plan has three main objectives: to redirect capital flows towards sustainable investment, to handle financial risk derived from green issues, and to promote transparency and a long-term orientation in economic activity and financial markets. In this context, the EU is aware of the importance of minimizing the impact of new financial stability risks stemming from environmental and social issues, and the key role to be played by the financial sector in achieving the environmental goals, as a large quantity of private capital is needed to bring about such change [39]. As a result, given the commitment of the EU to foster green energy through the development of a specific framework that promotes green energy production by reducing the specific risk associated with this type of sustainable investment, we expect that renewable companies in the EU are perceived as less risky, and are thus less affected by the pandemic than non-EU renewable companies. The EU support also positively affects the investor’s risk perspective since investments in renewable electricity are exposed to policy and institutional risks [28]. That bring us to our first hypothesis:

*H1*: *Renewable companies in the EU were less affected by the COVID-19 pandemic than non-EU renewable companies*, *both for the WHO announcement and the following national lockdowns*.

Investing in the renewable energy sector is considered risky, due to its high volatility [40], and the need for institutional support [11]. The weather-related nature of renewable electricity production causes uncertainty regarding the return on investment given the intermittency of the energy production [41]. In addition, investments related to renewable energy have more trouble reaching the break-even point, thus making it difficult to return the equity invested [42]. These obstacles to a successful investment together with the high capital expenditure required increases the perceived risk of the renewable energy sector [41]. Moreover, all the above risks make it difficult to effectively hedge this type of investment [43]. The consequences and difficulties that COVID-19 pandemic caused in the electricity sector are well documented in the literature: basically, uncertainty of the global demand and uncertainty in the investors’ return [44, 45]. The relationship between economic development and electricity is also showed in the literature [30, 46]. The comprehension of the magnitude of the impact of similar or related events to be drawn out from our research on energy sector, together with the economic consequences on sectors, countries and stock markets would help to deal with potential events in the future and increase the efficacy of public interventions, while unveiling potential opportunities [46]. Then, it is important to check and confirm whether the different types of energy, which having specific characteristics, had a different impact and reaction to the measures steaming from COVID-19 pandemic. That is why we aim to test our following hypotheses, in line with related literature that uses COVID-19 pandemic announcement and national lockdowns, by means of the unprecedented events we have chosen in our methodological approach. Due to the risky nature of renewable energy investment, we expect renewable companies to exhibit a larger negative impact due to COVID-19, both inside and outside the EU:

*H2a*: *Inside the EU*, *the renewable sector registered a greater decline than the traditional electricity sector*, *both for the WHO announcement of COVID-19 and the following national lockdowns*.
*H2b*: *Outside the EU*, *the renewable sector registered a greater decline than the traditional electricity sector*, *both for the WHO announcement of COVID-19 and the following national lockdowns*.

The arguments set out above emphasize the riskier profile of renewable energy investment together with the need for institutional support. It helps to understand why the EU’s support for this sector improves its risk-return profile [27, 47]. The EU is promoting a unified classification system or taxonomy that will provide clear guidance on activities qualifying as sustainable, and this EU taxonomy will be gradually integrated into the EU legal framework to provide more legal assurance. To mobilize private capital for sustainable investments, in addition to grants, the EU is significantly boosting its financial and technical support for sustainable infrastructure investments, such as the Connecting Europe Facility and the European Investment Advisory Hub [39]. To examine the need for institutional support, at the same time that with confirm the riskier profile of investing in the renewable energy sector, we compare the impact of COVID-19 inside and outside the EU and between renewable and traditional electricity companies. In our previous hypotheses, we aim to test whether the EU institutional context helped to reduce the uncertainty in the financial markets on renewable investments and the riskier perceived profile of renewable companies, even though traditional and renewable energy belong to the same sector. In our last hypothesis, we check whether the higher negative effect of COVID-19 on renewable companies compared to traditional, was softened by the influence of the EU framework. In this regard, we expect a lower negative effect for renewable companies compared to traditional companies, inside the EU in contrast to companies outside the EU, on the basics that the EU support helps to lower the perceived risk of investment in renewable electricity companies:

*H3*: *The higher negative effect of COVID-19 pandemic on renewable companies compared to traditional was lower for EU companies in comparison to Non-EU companies*, *both for the WHO announcement and the lockdowns*.

## Methodology

Following the related literature on stock price reactions to similar events, we use the event study method [7, 48, 49], taking the daily adjusted price to estimate abnormal returns (ARs) for each company *i* on day *t*.

To help ensure the robustness of our results, we use two alternative approaches, the market model and the Fama-French three-factor model.

The market model [50] is commonly used in the related COVID-19 literature [1, 3]. Following this model, the returns (from adjusted prices) for company *i* on day *t* are calculated as:

$$R_{i,t}=\alpha_{i}+\beta_{i}\;R_{m,t}+\varepsilon_{i,t}$$ (1)

where R_*i*,*t*_ are the returns for company *i*, and R_*m*,*t*_ are the market returns on day *t* in the estimation window (from 200 trading days before the event to 11 trading days before the event) for each event day (the event day is day 0). ε_*i*,*t*_ is the stochastic disturbance that meets the assumptions:

$$E(\varepsilon_{i,t})=0,\;\mathrm{VAR}(\varepsilon_{i,t})=\sigma_{i},$$ (2)

After estimating α_*i*_ and β_*i*_ based on the actual returns for every company, the expected return for company *i* on day *t* from t_0_ to t_1_ (t_0_ = -10, t_1_ = 10) is calculated as:

$$E(R_{i,t})={\hat{\alpha }}_{i}+{\hat{\beta }}_{i}R_{m,t}$$ (3)

Next, the ARs for company *i* on day *t* in the event window t_0_—t_1_ and the cumulative abnormal returns (CARs) are determined as follows:

$$AR_{i,t}=R_{i,t}‐E(R_{i,t})$$ (4)

$$CAR_{i}\;(t0,\;t1)=\sum_{t=t_{0}}^{t_{1}}{AR}_{i,t}$$ (5)

Finally, the average abnormal returns (AARs) for the companies in our sample and the cumulative average abnormal returns (CAARs) in the estimation window t_0_—t_1_ are:

$$AAR_{t}=\sum_{t=t_{0}}^{t_{1}}\frac{{AR}_{i,t}}{N}$$ (6)

$$CAAR_{i}\;(t_{0},\;t_{1})=\sum_{t=t_{0}}^{t_{1}}{AAR}_{t}$$ (7)

where *N* is the number of companies in our sample.

Second, we use the Fama-French three-factor model [51, 52], which controls for size and value, providing more explanatory power than the single-factor models [4, 48]. According to this second model, we calculate the adjusted price returns for company *i* on day *t* as follows:

$$R_{i,t}=\alpha_{i}+\beta_{i1}(R_{m,t}-R_{f,t})+\beta_{i2}\;SMB_{t}+\beta_{i3}\;HML_{t}+\varepsilon_{i,t}$$ (8)

where R_*i*,*t*_ are the returns of company *i* on day *t*, R_*f*,*t*_ are the risk-free returns on day *t* using the Euro Area 3-Month Bond Yield, R_*m*,*t*_ are the market returns for the S&P Europe 350 Index on day *t*, SMB_*t*_ is the difference in returns between small and large stock companies regarding market capitalization on day *t*, HML_*t*_ is the difference in returns between high and low book-to-market ratios on day *t*, α_*i*_ is the intercept of the relationship for company *i* and ε_*i*,*t*_ is the error term for company *i* on day *t*. In line with the literature, the risk-free return is the short-time interest rate of the sample and the market return is a market index that captures the general trend of the stock exchange, usually a global index like Dow Jones Global Index or an S&P 500 index [1, 4, 51, 52].

For each company, the coefficients ${\hat{\alpha }}_{i}$, ${\hat{\beta }}_{i1}$, ${\hat{\beta }}_{i2}$, ${\hat{\beta }}_{i3}$ were calculated using ordinary least squares linear regression for the estimation period, which is the period between 200 and 11 trading days before the event. Next, the ARs for company *i* on day *t* and the CARs during the estimation window t_0_—t_1_ are calculated as follows:

$$AR_{i,t}=R_{i,t}-({\hat{\alpha }}_{i}+{\hat{\beta }}_{i1}(R_{m,t}-R_{f,t})+{\hat{\beta }}_{i2}\;SMB_{t}+{\hat{\beta }}_{i3}\;HML_{t})$$ (9)

Finally, the CARs, the AARs, and the CAARs in the estimation window t_0_—t_1_ are estimated according to Eqs 5, 6 and 7.

To achieve more robust outcomes and ensure our results are not affected by companies with long trading suspensions, we use the last 200 trading days as our estimation window [1], from March 2019 to February 2020. Our event window is then defined as *t* ∈ (-10;10), where 0 represents the event day. We use 5 different intervals: (-10;0), (-5;0), (0;0), (0;5) and (0;10).

Finally, to verify the significance of our main results, we use the Wilcoxon t-test [53] as our data does not follow a normal distribution [4]. The Jarque-Bera normality test and Shapiro-Wilk test for normal data were used. Additionally, because of the correlation among financial markets in these types of events, we use the t-test for cross-sectional correlation of ARs [54–56], obtaining quantitatively and qualitatively similar results.

Our calculations were computed using Stata.

## Data description

In order to carry out our study, we have used data from different sources. We present the summary statistics of the variables in S1 Appendix at the end of the article.

First, to identify the listed electric utilities in Europe, we used Osiris, which is a database created by Bureau van Dijk. Following Zhang, Cao, Dickinson, and Kutan [11], we used GICS codes (Global Industrial Classification Standard) to identify European listed companies classified as traditional electricity producers (codes 55101010 and 55105010) as well as renewable electricity producers (code 55105020).

Second, as we are interested in the effect of COVID-19, we took the adjusted daily price (net of dividends). For every company, we extracted the data from the *yahoo finance* website. When the adjusted daily price was not available, we collected the data from the *investing*.*com* website, adjusting the collected daily price, net of dividends. With reference to the above GICS codes, we collected a total of 193 companies from Osiris, of which 64 were rejected because they did not have a minimum of 200 trading days [1]. As a result, we obtained 31 renewable and 98 traditional listed European electricity companies. However, for the event of lockdown, we had to eliminate Swedish companies since Sweden did not impose this restriction.

Third, to calculate the CAARs for every company, we need to know the starting date of every specific country lockdown. To that end, we used the information provided by the IMF in the website (International Monetary Fund). When said information was not available, we used the official website of the country.

Fourth, for our Fama-French three-factor model, we collected the daily yield for the Euro Area 3-Month Bond Yield from the European Central Bank, while the data for the three factors of the Fama-French model were collected from Kenneth R. French’s website.

Then, even though the WHO declaration of the COVID-19 pandemic occurred on 11^th^ March 2020 [3, 4, 15], we took the date as 12^th^ March. There are two reasons for this decision. First, the latter date is also commonly used in the literature [1]. Second, our study is about Europe and the WHO declaration was announced in the afternoon, when the European stock markets were closed. That is why we chose the day after the announcement as day zero. Finally, the group test we conducted showed that differences in the magnitude of the impact were significant, when comparing the announcement and national lockdowns effect, inside and outside the EU, for renewable and traditional companies.

Finally, we tested the possible association of CAARs between the two events to check whether the results might be influenced by it. We compared CAARs inside and outside the EU, for both models (market and Fama-French) both types of electricity and both events. Also we compared the difference between inside and outside the EU in the first event to the same difference for the second event, and for both types of electricity. The results show that in all the cases there is a significant difference in CAARs, so we can conclude that there is no association between CAARs in the announcement event and lockdown event.

## Results and discussion

Table 1 shows the resulting CAARs for the two events included in our study (the WHO’s announcement of a pandemic and when the national lockdown came into force in each country) in each event window. We also provide the CAARs for the traditional and renewable electricity sector for EU and non-EU countries.

**Table 1:** Cumulative average abnormal returns for the renewable electricity sector and traditional electricity sector in the EU countries and non-EU countries for WHO announcement and lockdown.

**Panel A: WHO announcement–Renewable electricity**					
CAARs for all 5 **non-EU** companies from the **renewable** electricity sector for WHO announcement					
*Event window*	***(-10;0)***	***(-5;0)***	***(0;0)***	***(0;5)***	***(0;10)***
Market model	-18.39%***	-19.75%***	-12.47%***	-14.19%***	-7.09%***
Fama-French model	-30.66%**	-29.89%**	-16.95%**	-20.64%**	-10.01%***
CAARs for all 26 **EU** companies from the **renewable** electricity sector for WHO announcement					
*Event window*	***(-10;0)***	***(-5;0)***	***(0;0)***	***(0;5)***	***(0;10)***
Market model	-16.69%***	-19.77%***	-9.10%***	-8.58%***	-2.98%***
Fama-French model	-26.36%***	-27.74%	-12.78%*	-13.97%***	-5.56%***
**Panel B: Lockdown–Renewable electricity**					
CAARs for all 25 **EU** companies from the **renewable** electricity sector for lockdowns					
*Event window*	***(-10;0)***	***(-5;0)***	***(0;0)***	***(0;5)***	***(0;10)***
Market model	-11.91%***	-12.36%***	-2.52%***	1.92%***	2.91%***
Fama-French model	-18.79%***	-15.20%***	-4.20%***	-3.24%***	-0.78%***
CAARs for all 5 **non-EU** companies from the **renewable** electricity sector for lockdowns					
*Event window*	***(-10;0)***	***(-5;0)***	***(0;0)***	***(0;5)***	***(0;10)***
Market model	-7.24%***	-10.90%***	-4.48%***	-0.34%***	3.41%***
Fama-French model	-20.14%***	-21.52%***	-9.16%***	-7.11%***	1.08%***
**Panel C: WHO announcement–Traditional electricity**					
CAARs for all 42 **EU** companies from the **traditional** electricity sector for WHO announcement					
*Event window*	***(-10;0)***	***(-5;0)***	***(0;0)***	***(0;5)***	***(0;10)***
Market model	-11.78%***	-12.22%***	-5.41%***	-3.22%***	-3.61%***
Fama-French model	-25.25%**	-23.63%**	-11.01%***	-10.71%***	-7.09%***
CAARs for all 56 **non-EU** companies from the **traditional** electricity sector for WHO announcement					
*Event window*	***(-10;0)***	***(-5;0)***	***(0;0)***	***(0;5)***	***(0;10)***
Market model	-22.04%***	-19.53%***	-7.27%**	-11.98%	-6.52%***
Fama-French model	-29.90%***	-26.45%***	-10.48%***	-15.99%***	-8.07%
**Panel D: Lockdown–Traditional electricity**					
CAARs for all 41 **EU** companies from the **traditional** electricity sector for lockdowns					
*Event window*	***(-10;0)***	***(-5;0)***	***(0;0)***	***(0;5)***	***(0;10)***
Market model	-8.00%***	-8.53%***	-1.65%***	1.32%***	-0.57%***
Fama-French model	-22.57%	-18.30%***	-4.84%***	-0.59%***	-1.71%***
CAARs for all 56 **non-EU** companies from the **traditional** electricity sector for lockdowns					
*Event window*	***(-10;0)***	***(-5;0)***	***(0;0)***	***(0;5)***	***(0;10)***
Market model	-1.70%***	-1.60%***	-0.07%***	6.03%***	5.81%***
Fama-French model	-0.80%***	2.13%***	0.95%***	6.29%***	7.86%***

*, **, and *** denote statistical significance at the 10%, 5%, and 1% level, respectively, using the Wilcoxon test. The full set of coefficients is available upon request.

**Fig 1:**
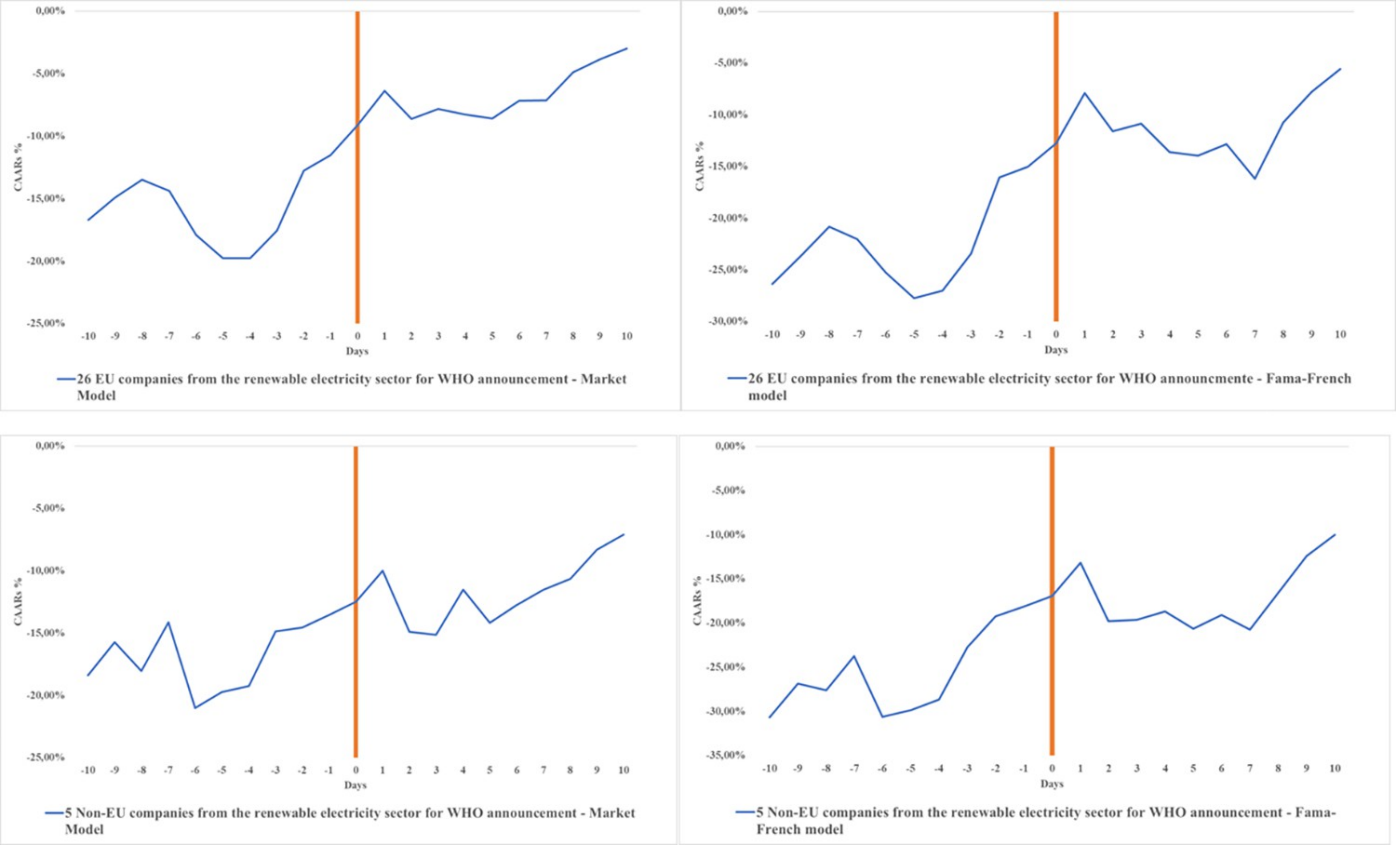


The results in Panel A exhibit significant negative CAARs meaning that the announcement of the pandemic by the WHO had an important effect on European renewable electricity companies, inside and outside the EU. COVID-19 reduced worldwide mobility, economic activity and thus electricity consumption [4]. To visualize the evolution of daily CAARs during the event window, we present several figures, displaying calculations made before and after day 0 (those shown in Table 1). In Fig 1, there was a downward effect 10 days before the announcement of the pandemic, reflecting a state of uncertainty developing in response to the events in China at the beginning of 2020. On day 0 for window (0;0) the CARs were in general more than half of the 10 days CAARs between window (-10;0) (e.g.-9.10% on the event day (0;0) and -16.69% for the prior ten days (-10;0)), which shows a significant negative effect of the WHO announcement on 12^th^ March 2020 on renewable electricity. The negative influence was felt over the course of 4 to 5 days after the announcement, as revealed by the dip in returns for the 26 EU and 5 non-EU companies.

**Fig 2:**
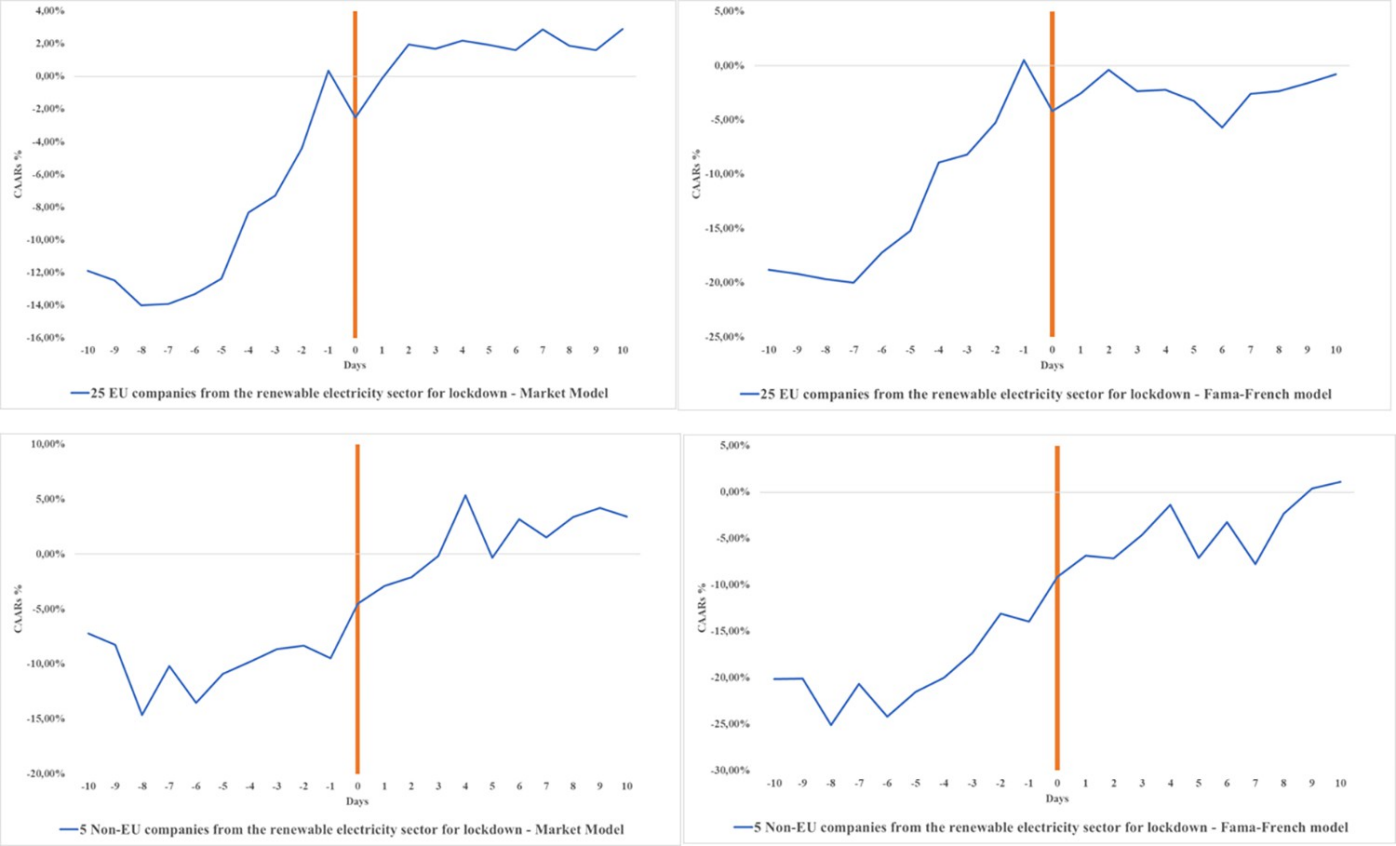


This significant negative effect is also displayed in Panel B for the effect of national lockdowns. In the same vein, looking at Fig 2, it seems to be the case overall that renewable electricity companies in the EU were less affected by the implementation of the lockdown than non-EU companies. The imposed lockdowns were less affecting for companies in the energy sector than the initial shock of the WHO announcement of the COVID-19 pandemic. After the lockdown was imposed, we observe positive CAARs, which indicates the start of a reversal in the market price, which was more positive for the EU companies that belong to the renewable sector. When we refer to the reversal effect, this turn implies that investors tend to initially overreact to “bad news” and then adjust their stance in the days that follow the shock [57]. This point is more relevant for the Fama-French model, which also takes into account size and values for companies.

However, in both events displayed in Panels A and B, the significant negative impact is lower for renewable electricity companies in the EU. This sector needs institutional support to make the investment profile less risky [27], and the EU’s aim in this regard is precisely to promote renewable energy; our first hypothesis is thus supported, confirming that the institutional support provided by the EU helps to reduce the uncertainty and risk perceptions of the renewable EU companies. As the EU has set itself the goals of boosting renewable energy consumption, reducing production costs, implementing assessment and monitoring mechanisms, and lessening its energy dependency, EU policies prioritize environmental protection and the increasing use of renewables [20]. Public policymakers can lower the risk of clean energy investments, supporting them by means of grants, funding, and tax incentives aimed at start-ups, private investors, while also encouraging corporations to participate in clean energy practices [42]. According to estimates from the European Investment Bank (EIB), there is an investment gap in the EU when it comes to energy saving, renewables and improving resource management [39], among other elements. That is why the EU, via the European Fund for Strategic Investments (EFSI), channels funds into energy, environmental and resource-efficient projects, together with technical assistance to support them.

To test hypothesis H2a, we compare the results for EU companies in Panel A with Panel C for the WHO announcement. The significant negative impact of COVID-19 was larger for EU renewable electricity companies, since investing in this alternative way of producing electricity is considered riskier than investing in traditional electricity [47]. For national lockdowns in the EU, we compare Panel B with Panel D, with mostly similar results. Therefore, hypothesis H2a is supported. To test hypothesis H2b for European electricity companies outside the EU, we compare Panel A with Panel C, and Panel B with Panel D, for the WHO announcement and national lockdowns, respectively. As with hypothesis H2a, the results support hypothesis H2b; namely, that the significant negative effect was larger for renewable electricity companies. Despite the growing importance of electricity generation from renewables, investing in these types of projects is typically subject to serious risks, which affects potential investors’ decisions [40]. Some countries have started to cut grants for renewable projects, and a funding gap is expected to occur for this type of investment [39, 43]. Moreover, renewables tend to exhibit substantial price volatility [58], raising the risk of whether they will provide the expected initial return. Recent studies show that investments in clean energy technology are more likely to fail and have a low return on capital compared, for instance, to investment in medical and software technology [42]. Furthermore, renewable energy investments are characterized by needing high levels of capital expenditure, which, associated with the high probabilities of failure, make it difficult to break even. Moreover, weather dependency means the investment is hard to hedge, making it even riskier. In addition to the intrinsic risk of renewable electricity, COVID-19 disrupted the renewable components supply chains creating delays in the operating of renewable energy facilities [59].

To test our third hypothesis, we proceed as follows. For our two events, we compare the difference in the significant negative impact between renewable and traditional companies, both inside and outside the EU (Panel A and Panel C). We choose window (0;0) as an example. The significant negative impact for EU renewable electricity companies was -9.10%, whereas for EU traditional companies it was -5.41%. The difference between the two was -3.69%, reflecting a larger negative impact for EU renewable electricity companies. The significant negative impact for non-EU renewable companies was -12.47%, whereas for non-EU traditional companies it was -7.27%. The difference was then -5.20%, a larger negative impact for the non-EU renewable companies. As we find a larger difference between renewable and traditional companies for non-EU electricity companies (-5.20% versus -3.69%), our third hypothesis is supported. In the same vein, for the event of national lockdowns, we compare Panel B and Panel D, obtaining similar results. The EU provides a specific framework that helps to reduce the perceived risk of investments in renewables, since legislation and public administration support are essential factors for the development of green investments [26]. Finally, in countries that are in Europe but outside the EU, the choice to invest in renewable energy is not as compelling as in the EU, since many of the former are, with Russia leading the way, the main exporters of fossil energy sources worldwide [22, 60].

## Conclusions and policy implications

By means of the event study method, our study analyses the effect of COVID-19 on the electricity sector, estimating and examining the CAARs of European listed electricity companies both inside the European Union and outside. We split our companies into subsectors of renewable and traditional electricity production. In the event study approach, we use two important events related to COVID-19, which are the World Health Organization’s announcement of the pandemic and the implementation of national lockdowns for European countries.

Our results show that renewable electricity companies had a larger significant negative impact, showing more negative CAARs than traditional companies, in both events, announcement of the pandemic and national lockdowns. COVID-19 reduced mobility worldwide, slowed global economic activity, and cut electricity consumption, thus generating uncertainty in financial markets [4]. Since renewable electricity companies represent a risky investment, the negative effect was larger. Compared to traditional electricity, renewables show price volatility, a high rate of failure, high level of capital expenditure, trouble breaking even, and an intermittent production, all of which increases the risk of whether they will provide the expected return [42, 43, 47, 58]. Additionally, COVID-19 affected the supply chain of specific components, leading to delays in the functioning of renewable energy projects. After the lockdown was imposed, there was a start of a positive reversal in the market price, which was more positive in the EU renewable companies. When we refer to a reversal effect, this turn implies that investors tend to initially overreact to “bad news” and then adjust their stance in the days that follow the shock. This was evident in our study from day 0 to 10 days after the event.

Furthermore, our findings show the need of institutional support in renewables investment. While the negative significant impact was larger on renewable electricity companies than on traditional electricity companies, this difference was smaller for EU companies in both events. Our findings are in line with previous empirical evidence showing that the renewable electricity sector needs support from public administrations to reduce the investment risk and change the risk-return association [27, 42, 61]. The EU aims to support and promote renewable energy, prioritizing the use of renewable electricity [20, 39]. As such, it has developed different lines of action: promoting a taxonomy of clean energy investment to provide a legal framework, thereby boosting investors’ confidence; mobilizing funds to fill the renewable investment gap in the EU; and providing technical support for sustainable investments.

The overall findings of our study have important implications for policymakers. As stated before, the EU presents a clear support of renewable energy, the “European Green Deal”, materialized through strategic policies that involve substantial economic investments (such as the Action Plan), at the same time that provides a supportive and stable financial frame (via the EU’s Capital Markets Union and the Euro as common currency). We have compared the effect of two unique unprecedented events in renewable and traditional sectors, distinguishing between companies listed in EU countries (which provides an institutional framework and the use of the same currency) and Non-EU countries. This empirical strategy allows us to test whether the effect of the EU context influences the uncertainty of investors represented in the financial markets by the stock prices return. We corroborate that those investments related to clean or renewable energy suffer from specific risks. Together with the intrinsic risks described above (volatility, breaking even, uncertainty of the return, high capital expenditure) which pose major challenges with respect to investment optimization [28], these investments also present policy and regulatory risk, since policymakers and institutional support are key determinants of the successful development of this sector [26]. Policymakers should bear in mind that expectations about future industries and markets play an important role in investments decisions [62]. Investors need a stable regulatory framework linked to long-term sustainable objectives that help them to allocate private equity with confidence [24]. In this vein, policymakers need to develop policies aimed at mobilizing financial resources and reducing uncertainty in the regulatory environment, as the EU is developing its sustainable infrastructure programme. Limitations affecting financial support for new renewable energy sources, especially the unpredictable or limited time period for this support, can be a considerable policy risk for renewable electricity companies; policymakers must take this into consideration as the pandemic is not yet over.

Our study shows to policymakers the higher financial sensitivity of investors in renewable energy via stock prices and financial market returns. Furthermore, how they can foster, encourage, and furnish stability to this sector, even in unparalleled shocks as the COVID-19 pandemic worldwide declaration and the consequent national lockdowns, by developing an institutional context that provides, among others, long-term regulatory certainty and funding support. The crisis generated by COVID-19 has revealed the vulnerability of industrial countries to supply chain issues and the critical role played by energy. EU policymakers should thus reconsider their energy policy in terms of protecting their strategic resources from supply shortages. In this vein, policymakers from other countries may consider the implications of our findings in their policy-decision process regarding how to promote renewable energy in their country in line with the EU strategy. Institutional policies such as providing a supportive and stable financial frame (like the EU’s Capital Markets) and plans that involve substantial economic investment (like the Action Plan in the EU) could be used a reference or guidelines. This supportive framework would help to create a stable investment framework that would encourage positive investment expectations, reduce uncertainty together with a lower investor’s sensitivity to the effect of unexpected events in their stock prices and returns, as shown in our study. Given the arising strategic importance of this type of energy in view of the critical geo-political events taking place during 2022 [63, 64] it becomes clear the need of this institutional support.

An important point for future research is to analyse the EU energy policy implications in comparison to other developed or developing regions (China, US, Canada) taking into consideration that the Green New Deal sets principles to fully decarbonizing developed economies while also reducing inequality. Another interesting future line of research would be to explicitly calculated risk measures (such as volatility, illiquidity discount, etc.) for both type of investments inside and outside the EU or in similar economic areas that foster green investments. These findings would help to corroborate the need of institutional support in this type of investments.

## Supporting information

S1 Appendix Descriptive statistics. (DOCX)
