# Supplementary material for: A comparative study on renewable and traditional electricity: The influence of the European Union framework and the impact of COVID-19
Source: PLoS One. 2022 Nov 21;17(11):e0277088. doi: 10.1371/journal.pone.0277088 (PMC9678253; doi:10.1371/journal.pone.0277088)
Supplement: S1 Appendix — (DOCX) [file pone.0277088.s001.docx]

**Appendices**

**Appendix 1. Descriptive statistics**

|  |  |  |  |  |  |
| --- | --- | --- | --- | --- | --- |
| Variables | N | mean | std | min | max |
|  |  |  |  |  |  |
| MKT | 344 | 0.002% | 1.44% | -12.30% | 8.15% |
| SMB | 336 | -0.02% | 0.69% | -3.59% | 5.54% |
| HML | 336 | -0.12% | 0.92% | -4.71% | 3.19% |
| Rm-Rf | 340 | 0.01% | 3.12% | -20.40% | 12.60% |
| Rf | 340 | -0.01% | 2.46% | -10.70% | 12.60% |

Notes: This table presents the descriptive statistics of the main variables. N is the number of observations, mean is the average of the returns for each company and indicator, std is the standard deviation and min and max are the minimum and maximum of the returns. MKT is the abbreviation for the S&P Europe 350 Index, SMB for the small and large stock companies, HML for high and low book-to-market ratios, and Rf for the risk free rate, and Rm-Rf the abbreviation for the difference between the market return and the free risk rate.
